# Mobile direct observation of therapy (MDOT) - A rapid systematic review and pilot study in children with asthma

**DOI:** 10.1371/journal.pone.0190031

**Published:** 2018-02-05

**Authors:** Michael D. Shields, Fahad ALQahtani, Michael P. Rivey, James C. McElnay

**Affiliations:** 1 Centre for Infection and Immunity. School of Medicine, Dentistry and Biomedical Sciences, Queen’s University Belfast, Belfast, United Kingdom; 2 Clinical and Practice Research Group, School of Pharmacy, Queen’s University of Belfast, Belfast, United Kingdom; 3 Department of Pharmacy Practice, College of Health Professions and Biomedical Sciences, University of Montana, Missoula, Montana, United States of America; TNO, NETHERLANDS

## Abstract

We describe, for the first time, the use of a mobile device platform for remote direct observation of inhaler use and technique. The research programme commenced with a rapid systematic review of mobile device (or videophone) use for direct observation of therapy (MDOT). Ten studies (mainly pilots) were identified involving patients with tuberculosis, sickle cell disease and Alzheimer's disease. New studies are ongoing (ClinicalTrials.gov website) in TB, stroke, sickle cell disease, HIV and opioid dependence. Having identified no prior use of MDOT in inhaler monitoring, we implemented a feasibility study in 12 healthy volunteer children (2–12 years; 8 females and 4 males) over a period of 14 days, with twice daily video upload of their 'dummy' inhaler use. Two children uploaded 100% of the requested videos, with only one child having an inhaler upload rate of <75%. The quality of uploaded videos was generally good (only 1.7% of unacceptable quality for evaluation). The final aspect of the research was a pilot study using MDOT (6 weeks) in 22 children with difficult to treat asthma. Healthcare professionals evaluated inhaler technique using uploaded videos and provided telephone instruction on improving inhaler use. The main outcomes were assessed at week 12 post initiation of MDOT. By week 5, all children still engaging in MDOT (n = 18) were judged to have effective inhaler technique. Spirometry values did not vary to a significantly significant degree between baseline and 12 weeks (P>0.05), however, mean fraction of exhaled nitric oxide (FeNO) values normalised (mean 38.7 to 19.3ppm) and mean Asthma Control Test values improved (13.1 to mean 17.8). Feedback from participants was positive. Overall the findings open up a new paradigm in device independent (can be used for any type of inhaler device) monitoring, providing a platform for evaluating / improving inhaler use at home.

## Introduction

Asthma is a health problem affecting approximately 253 million people worldwide and it is the most common non-communicable illness in children [1]. Many studies have reported that the majority of patients (50%-80%) fail to use effective inhaler technique and this inadequate inhaler use is one of the reasons for the high prevalence of uncontrolled asthma [2–4]. Moreover, it has been noted that most patients overestimate their inhaler use capability and are not aware of making mistakes during inhaler use [5,6]. Although inhaled corticosteroid therapy (ICS) is central to asthma management, some patients do not use their treatment as prescribed and indeed the average medication adherence to inhaled corticosteroid (ICS) in children is documented to be around 50% [7,8]. To achieve optimal outcomes, it is not only imperative that children adhere to the regular use of their ICS (normally twice per day) but that they adhere to correct inhaler technique, allowing the medicine to penetrate into, and be deposited deep within, the lungs [5].

Non-adherence to medication in children with asthma can have various negative consequences such as frequent clinic visits, disease exacerbation and hospital admission, all of which lead to an increased cost of care [4,9]. Indeed, a review of childhood asthma deaths in SE England showed that medication non-adherence was a contributing factor in more than 50% of cases, including patients with milder disease [9]. A multicentre clinical trial in the USA was designed to determine what medication was best to add in children with severe asthma already receiving ICS and long acting beta agonist (LABA) treatment. In this latter study the run-in period was not fixed and children were only randomised to therapy after they had demonstrated good adherence, including good inhaler technique. Interestingly, the trial was cut short due to an inability to recruit an adequate number of children. Patient improvement while under close supervision during the run in period was the main reason for the lack of patient recruitment [10] showing that intense monitoring and improved adherence led to the asthma becoming better controlled.

New therapies for children with uncontrolled asthma such as anti-IgE therapy (omalizumab) are very expensive and thus in the UK most large paediatric respiratory centres have set up specific ‘problem, severe asthma’ clinics. These regional centres should have the ability to confirm the asthma diagnosis, ensure that concomitant conditions are treated and, most importantly, confirm that the children still have symptoms after a period of optimised therapy (including good adherence to ICS and good inhaler technique). With the exception of directly observing therapy, there is currently no adequate generalizable method or tool to determine whether the therapy has been optimised i.e. that enables the doctor to know that the child has used his/her inhaler regularly and correctly. This involves a healthcare professional regularly visiting the patient at home or school for a period of 6 to 12 weeks which is very costly and labour intensive. There is a pressing need for a convenient, simple tool that could mimic this direct observation of therapy (DOT) without the staff and travel costs.

DOT has been promoted by the WHO to enhance the adherence of patients with tuberculosis (TB) [11,12]. A new development within the DOT arena, linked to the trend of the increased use of mobile IT devices within the healthcare field, has been the introduction of mobile DOT (MDOT). This emerging approach to assessing treatment adherence, involves a patient recording a video of their treatment administration at home on a mobile device (e.g. on their mobile phone, tablet or videophone) and then sending it electronically for observation by a healthcare provider [13,14]. MDOT has the potential to significantly reduce the cost associated with traditional DOT and has been shown to be effective in TB patients [14] and in patients with sickle cell disease [13] in whom good medication adherence is imperative. For example, a smartphone application was developed specifically for applying a MDOT approach to enhance paediatric hydroxyurea medication adherence in children with sickle cell disease [13]. This latter approach resulted in a median adherence of 93.3% over a six month period.

The aim of the present study was threefold: (a) to carry out a rapid systematic review of the use of MDOT within healthcare, and in particular its prior use in asthma management, (b) to carry out a healthy volunteer study to assess the feasibility of using MDOT to monitor / evaluate inhaler use in children and (c) perform a pilot study of the use of MDOT in children with difficult to control asthma.

## Methods

### Rapid systematic review

The methodological protocol for this rapid review followed the guidance outlined by Khangura *et al*. [15]. The literature search was carried out in April / June 2015.

All the published studies (English language) which utilised a MDOT technique were included regardless of the age or disease condition of the participants. For the purposes of this review, any study which used a video phone, smartphone or other mobile device to record a video of treatment administration at home, in which the video could be remotely observed by a healthcare provider, was included.

Six different databases i.e. MEDLINE; Scopus; Web of Science; International Pharmaceutical Abstracts (IPA); PubMed and EMBASE were rigorously searched using the following subject headings (MeSH terms) and keywords:

("video recording" OR smartphone /or handheld device OR Telemedicine / or mobile video OR Social media /or mobile applications) AND (directly observed therapy / or "direct observed" OR "directly observed" OR "direct observation"). The reference lists in each study identified were also searched for relevant studies.

In addition, an electronic online search was carried out via Google^®^ in order to search more widely for undiscovered studies, grey literature and conference abstracts. The ClinicalTrail.gov website was also searched for new / ongoing clinical trials that were relevant to the review.

Having removed duplicates, full-text versions of the research articles were obtained. Studies that were not available in full-text version format were excluded (n = 2). The quality of the articles were assessed by three of the authors (FA, JM and MR) using the Downs and Black [16] instrument (as modified by Eng *et al*.) [17]. This tool has been shown to be valid and reliable in assessing randomised and non-randomised studies and contains 27 items arranged in four sub-scales, i.e. reporting, external validity, internal validity and power (score range 0–28; excellent 26–28; good 20–25; fair 15–19; poor <14). The outcome of the review is presented in the results section of this paper. No prior use of MDOT approach in asthma management was identified.

### Feasibility study–healthy volunteer children

The aim of this aspect of the research programme was to evaluate the feasibility of using the MDOT approach to capture the use of ‘dummy’ inhalers in a small cohort of healthy volunteer children (n = 12) ahead of carrying out a pilot clinical study in paediatric patients with difficult to control asthma. A simple MDOT platform was developed which was compatible with all brands of smartphone, tablets or other devices that could be connected to the internet, and allowed video recording and encrypted transmission to a secure, password protected University server.

The study protocol was approved by the School of Pharmacy Ethics Committee (School Ref: 014PMY2014). A convenience sample involving children of staff working in health and social science departments in Queen’s University was recruited, i.e. via an email invitation, staff members were invited to take part in the study if they had children between the ages of 2 and 12 years old who did not suffer from asthma and who did not use an inhaler for any other condition. In addition, to be enrolled on to the study, parents (i) had to have a smartphone, tablet or other mobile device that could capture video clips and connect to the internet, (ii) had to have internet access at home and (iii) were required to provide written informed consent to their child’s participation. Children over 6 years old were required to provide their assent to join the study. Once enrolled in the study, the MDOT platform was set up on each parent’s /guardian’s smartphone, tablet (or other mobile device) to allow video capture and encrypted transmission. Parents/ guardians (and their children) were instructed in correct inhaler use and were asked to facilitate the capture and upload of two video recordings daily (morning and evening) of the use of the dummy inhaler by their child over a period of two weeks. For children up to the age 6 years old, a mask and holding chamber was used with the dummy inhaler. In general the parents/guardians ‘administered’ the inhaler for children up to 6 years old while children 6 years old and above were asked to use the dummy inhaler by themselves. A member of the research team (FA) reviewed video uploads on a daily basis, through password protected access to the repository and followed up with the parent/guardian by telephone if two consecutive days of video uploads were missed.

When the two week period of video collection was completed, parents/guardians and children over 6 years old were invited to take part in a joint interview to provide their views and experience regarding the use of the technology. A topic guide was developed to guide the interviews. During interviews with children, simplified language appropriate to their ages was used. All interviews were audio recorded and transcribed in full. The software package NVivo (IQSR international, version 10) was used to store and manage / analyse interview transcripts.

Having completed all video collections, an assessment of video quality was undertaken to determine whether the videos were of good enough quality to assess inhaler use. Five videos were randomly selected (random number generation; www.random.org) for each child i.e. 60 videos in total for the 12 participating children. The video quality was categorised as: (i) acceptable video quality, when the video had good clarity, framing, image stability and would be suitable for assessing the various steps required for scoring inhaler technique, (ii) marginally acceptable video quality was recorded when there was some aspect that affected video clarity or resolution but a general assessment of inhaler technique would still be possible and (iii) Unacceptable video quality was recorded when the video image was not clear and it would not be possible to score the inhaler steps (e.g. poor lighting) or when two or more steps in inhaler use were not clearly visible in the video recording. This evaluation was performed independently by each member of the research team (n = 4)

The primary outcome measures for this feasibility study related to the connectivity and usability of the MDOT system and therefore primarily related to (a) the number of days in which videos were loaded and (b) the quality of uploaded videos. Barriers to using the MDOT platform and to loading the videos on to the repository developed specifically for the study were determined via the interviews.

All numerical and categorical data collected during the study were analysed using descriptive approaches while the interview transcripts were analysed qualitatively (thematic analysis) [18,19].

### Pilot study in children with asthma

Having achieved encouraging results in the feasibility study, attention turned to a pilot study in children with difficult to manage asthma. The study protocol was granted a favourable ethical opinion (reference number: 14/NI/1099) from the Research Ethics Committees Northern Ireland (ORECNI) and is registered with ClinicalTrials.gov PRS (reference number: NCT03248895). Parents / guardians and their children were recruited at outpatient appointments at two sites in the Belfast Health & Social Care Trust i.e. Royal Belfast Hospital for Sick Children (RBHSC) and the Trust’s Community Asthma Clinic.

#### Study design

A randomised intervention trial design was used in which participants were randomised to receive either an immediate (IM) or a delayed (DE) MDOT intervention. Randomisation was restricted [20] based on two factors i.e. age (categorised as ‘young children’ aged 2 to 5 years; ‘children’ aged 5 to 12 years; ‘young people’ aged 12 to 16 years) and patient gender.

Participants allocated to the IM group participated in the MDOT intervention for the first 6 weeks of the study. Those participants allocated to the DE group commenced the MDOT intervention after a 6 week “intervention-free” period, with usual Asthma Clinic care up to that point. The original plan was to recruit 72 children (36 per group). Although the MDOT approach was well accepted by clinicians (including asthma nurses), parents and patients, due to research staffing pressures a lower sample size was achieved (n = 24). The main focus was therefore on combined data at study baseline and endpoint, i.e. baseline and 12 weeks post initiation of the MDOT intervention.

#### Recruitment of participants

The parents/guardians of children and young people with asthma (aged from 2 to16 years) who had continuing symptoms of partially controlled or uncontrolled asthma, despite receiving specialist asthma clinic care, were invited to participate in the research i.e. children who had asthma symptoms despite being prescribed ICS (> 400 mcg/day budesonide equivalent for children < 5 years, or 800 mcg/day budesonide equivalent for children > 5 years) and a second line therapy such as a long acting beta agonist (LABA), leukotriene receptor antagonist (LTRA) or theophylline [21]. Access to a suitable mobile device and internet connection at home, as for the feasibility study, was also required. Subjects were only included in the study after obtaining written informed consent from their parents/guardians and assent from children older than 6 years. Recruitment of participants began in August 2015 and follow-up completed by the end of September 2016.

All subjects (parents and children as appropriate) were trained, on proper inhaler technique (by a respiratory nurse) using the Teach-to-Goal (TTG) approach [22] and received standard-of-practice asthma education and management in the Asthma Clinic prior to study enrolment i.e. educated about the role of medications in asthma management, the importance of regular preventer therapy and were able to demonstrate good inhaler technique.

#### Intervention and data collection

Before the study intervention period was initiated in a given subject, the participant and/or parents/guardians were trained in use of the MDOT platform. Children aged ≥ 8 years were asked to use the MDOT approach independently while parent/guardian assistance was recommended for younger children. Usual asthma treatment for each participant was used throughout the study. Regarding inhaler use, in general children aged 5 years or older administered the medication themselves while a parent/guardian was responsible for inhaler administration in patients 2 to 5 years of age, however, parent/guardian administration was acceptable if that was usual asthma care in any age of child.

Participants (or parents/guardians of participants) were asked to capture a MDOT video twice daily as in the feasibility study. A member of the research team evaluated the MDOT uploads daily through password protected access to the University based digital repository and followed up with parents/guardians and/or participants by telephone if two consecutive days of video uploads were missed. The asthma clinical team (consultant/nurse) was notified about children identified with poor inhaler technique; they viewed the videos and contacted the participants by telephone to provide advice on improving inhaler technique.

#### Outcome measurements

The range of measures at baseline were as follows:

1. Participant demographics and relevant clinical data including laboratory data and comorbid conditions.
2. Clinician assessment of asthma severity and degree of disease control i.e. controlled, partially controlled, or uncontrolled [23].
3. Asthma medication profile and any changes made at the clinic visit.
4. Number of asthma attacks, oral corticosteroids courses, and emergency attendances over the previous year.
5. Spirometry measurements (FEV_1_, FVC, FEV_1_/ FVC)
6. Fraction of exhaled nitric oxide (FeNO)
7. Self-reported Medication Adherence Report Scale (MARS)–appropriate scales for parent/guardian and child if 9 years of age or greater [24–26].
8. Interview-administered Pediatric Asthma Quality of Life Questionnaire (PAQLQ) if child >9 years of age, and the Pediatric Asthma Caregiver Quality of Life Questionnaire (PACQLQ) [27].
9. Interview-administered Asthma Control Test (ACT) or Childhood Asthma Control Test (C-ACT) [28, 29] for children aged to 12 to 16 and 4 to 11 years respectively.

At six weeks and 12 weeks post commencement of the MDOT intervention, outcome measures ii to ix were repeated during return visits to the clinic.

#### Assessment of adherence

In addition to the MARS questionnaire approach to assess adherence to ICS (see above), GP prescribing records for the 12 months prior to study commencement were collected and analysed using the medication refill adherence (MRA) approach described by Hess *et al*. [30] to ascertain baseline adherence. Adherence to MDOT video uploads was also assessed during the 6 week MDOT intervention in all participants. A patient was classified as adherent if the MRA value was ≥80% to <120% and non-adherent if MRA< 80%. If the MRA was found to be >120% this was considered as oversupply. This classification method was utilised by previous researchers [31–34]. Patients were classified as adherent if the MARS (parent; child) score was ≥ 80% of the maximum score achievable in the respective versions (parent and child) of the MARS questionnaire.

#### Assessment of asthma severity and inhaler technique

Clinician assessment of participant asthma severity was defined according to accepted guidelines and asthma control was categorised as uncontrolled, partially controlled, or controlled [23]. Patient inhaler technique was evaluated by reviewing each video uploaded in the first 2 days of the intervention period and then via a weekly review of videos uploaded to the repository. Inhaler technique was categorised on a three-point scale as effective, partially effective or poor [35]. Inhaler technique evaluation was based on the individual steps relevant to the type of inhaler being used by individual children (manufacturer administration directions). Effective technique was recorded when the participant met all relevant criteria for their inhaler type. Partially effective technique was recorded when errors in technique were observed but it was thought that the participant would receive some medication. Poor inhaler technique was recorded when critical errors in technique occurred and it was considered unlikely that any medication would be inhaled [35,36]. Sample videos was reviewed and scored by two members of the research team (FA; JM).

#### Feedback questionnaire

Parents/guardians were asked to complete an ‘open question’ feedback questionnaire to provide their views and experience with the use of the technology at the end of intervention period. Feedback questionnaires were sent to each participant by post and each participant was contacted by telephone as a reminder if the questionnaire was not returned within a two week period. Two feedback questionnaires were used: one for participants who completed the 6 week period of using MDOT and the second for participants who consented to take part but did not upload videos or who uploaded videos for a shorter period that the 6 weeks requested.

#### Data management and analysis

All data were coded as appropriate and entered into a study database (SPSS^®^ version, 21, USA). All categorical data collected during the pilot study were analysed using percentages and proportions. Continuous variables were described using mean and standard deviation (SD) or if skewed median and interquartile range (IQR). Standard statistical methodology (t-test and Mann-Whitney U test) were used to compare outcome data.

## Results and discussion

### Rapid systematic review (RSR)

The methodological approach used in this review mirrored the approach used by Khangura *et al*. [15]. Several published RSRs have used this latter methodology [37–39]. This review technique provides a quick approach to evaluate current evidence on a selected topic [40].

**Fig 1:**
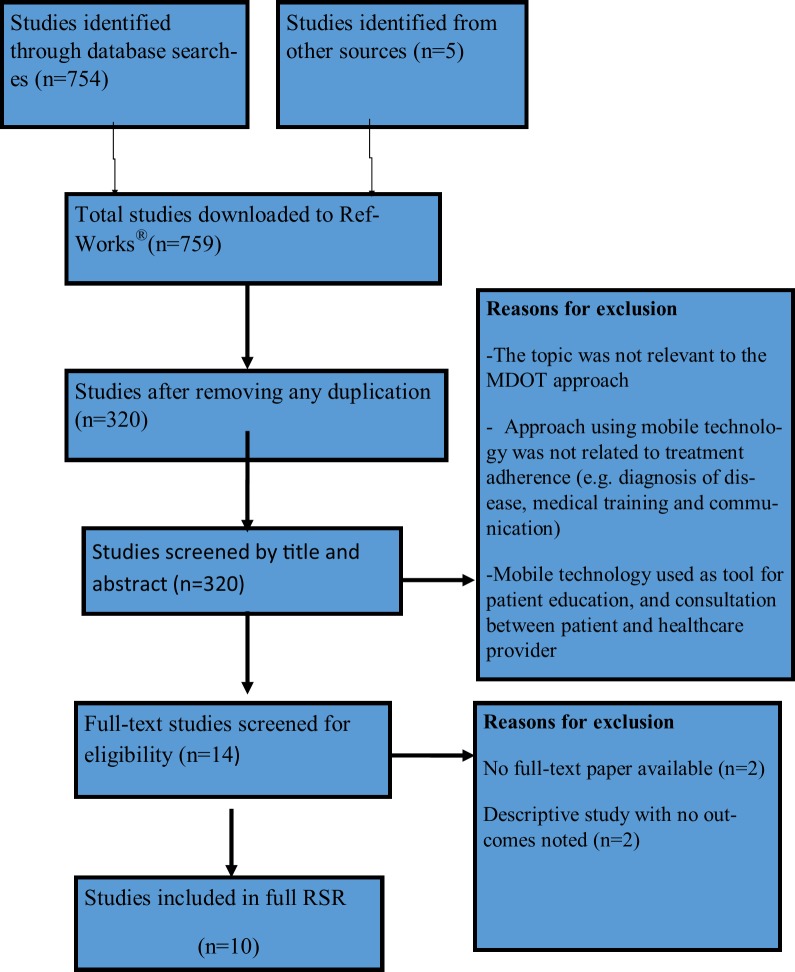
Study extraction and selection process in rapid systematic review (RSR).

Fig 1 presents the number of studies at each stage of the selection process, including reasons for study exclusion. A total of only10 studies remained for inclusion in the main aspect of the review. Only non-randomised studies on the subject had been published. The highest score achieved for any of the studies, using the modified Downs and Black instrument [16,17], was 20 (out of a maximum possible score of 28), and the lowest score was 5. The median score for all the articles was 11.3 which equates to poor quality. It can be concluded that MDOT studies performed to date have suboptimal design / reporting and therefore results need to be considered with caution.

All the published studies were conducted to assess treatment adherence in tuberculosis, with the exception of two studies: one involving in the treatment of children with sickle cell disease and the second involving dementia. The majority of the research was carried out in the USA (six studies), while two studies were performed in Australia. Single studies were performed in several other countries: Kenya, Mexico and Canada. Relevant information from the studies were systematically extracted and are summarised Table 1. Six of the studies were described by their authors as pilot studies. All these pilot studies demonstrated that utilising technology for monitoring adherence via a MDOT approach was feasible, economical and practical, including in one study when used by patients with mild dementia [13,41,45,47,49]. The majority of the studies reported that the IT supported approach has the potential to improve treatment adherence, with rates of adherence of over 80% reached. Two of the pilot studies involved paediatric patients (hydroxyurea treatment and tuberculosis treatment).

**Table 1:** Summary of the characteristics of the study included in the rapid systematic review (RSR).

Author, Year, Country	Title and Journal	Device used for recording video of treatment and study duration	Method: intervention/ sample size /age group of participants	Aim of study	Findings	Conclusions
Garfein *et al*., 2015. [41] **USA and Mexico**	Feasibility of tuberculosis treatment monitoring by video directly observed therapy: a Binational pilot study **International Journal of Tuberculosis and Lung Disease**	Smartphone. The recording lasted for approximately 5 months.	Pilot study recruited 52 patients (18–86 years) from San Deigo (US) and Tijuana (Mexico). Recorded video of treatment administration and submitted to be seen by health worker.	To investigate, using videophone recording, adherence to TB treatment in two countries, US and Mexico. Also, examined the extent of patient satisfaction.	The adherence rate was comparable in the two nations; 90% (San Deigo) and 96% (Tijuana). Mobile use in this study was highly acceptable.	Video directly observed therapy is a satisfactory approach and can be used to assess adherence in rich or poor resource countries.
Mirsaeidi *et al*., 2015. [42] **USA**	Video directly observed therapy for treatment of tuberculosis is patient-oriented and cost-effective. **European Respiratory Society.**	Mobile phone or laptop. The majority of participants used this approach up to 4 months (64%) while 24% used it up to 8 months.	Retrospective study; 20 patients used DOT method and were compared with 11 patients who used videophone assessed DOT (19–64 years old).	Assessment of the cost effectiveness of observation and the level of satisfaction with the videophone DOT approach.	All videophone patients (n = 11) completed the study. 9 patients used a mobile phone or a laptop for observation. Recorded rate of adherence was 97%.	Observation via mobile phone was feasible and had a high tendency to improve adherence and save costs.
Creary *et al*., 2014. [13] **USA**	A pilot study of electronic directly observed therapy to improve hydroxyurea adherence in paediatric patients with sickle-cell disease. **Pediatric Blood and Cancer**	Smartphone or computer. The study duration was 6 months.	Electronic observed therapy method was used to measure adherence of hydroxyurea in 15 patients (1–22 years) with sickle cell disorder.	To study the feasibility of electronic adherence monitoring of hydroxylurea in children via recording treatment using mobile phone application or computer.	Fourteen children completed the pilot study. The median of treatment adherence was 93.3%. The approach had a high rate of acceptance.	Recording medicine taking via electronic device was feasible, highly acceptable and could be utilised to promote adherence.
Gassanov *et al*., 2013. [43] **Canada**	The use of videophone for directly observed therapy for the treatment of tuberculosis **Canadian Journal of Public Health**	Video phone. The trial duration was five weeks.	Pilot study involved 13 patients who successfully adhered to DOT at clinic. Patients supplied with a video enabled telephone to use at home, with network connection to clinic for observation remotely.	To evaluate video DOT flexibility in the delivery of video enabled DOT method at home.	Videophone was convenient and had flexibility for observation. The rate of adherence for patients attending clinic (DOT) method (98%) was comparable to patients using videophone method at home (98%).	Recording by videophone at home had comparable outcomes to DOT at clinic.
Wade *et al*., 2012. [44] **Australia**	Home videophones improve direct observation in tuberculosis: A mixed methods evaluation. **Public Library Of Science (PLOS ONE)**	Video phone. The video recording was for a 4 month period.	Retrospective study using qualitative and quantitative approaches. 58 patients used videophone service while 70 patients were observed personally at home. The study recruited children and adults. Participant’s age ranged between 19 and 60 years.	To evaluate the feasibility and economic benefit of using video phone observation at home compared with direct clinic observation of administration of tuberculosis therapy.	Lower percentage of missed observations by the video phone group (12%) compared with 31.1% in the direct clinic observation group. Fewer staff and less time required for video phone group.	Directly observing TB patients via video phone was more effective than directly observing them in the clinic, and led to cost savings. The video phone service was associated with more flexibility, patient acceptability and efficiency in relation to healthcare professional time.
Hoffman *et al*., 2010. [45] **Kenya**	Mobile direct observation treatment for tuberculosis patients: A technical feasibility pilot using mobile phones in Nairobi, Kenya. **American Journal of Preventive Medicine**	Mobile phone. study carried out for 4 week period (five days/ week)	Pilot study involved 3 health care providers and 11 patients (all patients <29 years except for one patient over 40 years old). Recorded video of TB medicine administration and submitted via mobile phone.	To study the feasibility of observing TB patient administering medication remotely via the mobile DOT approach.	All participants completed the study. 8 patients preferred to use mobile phone rather than coming to hospital for observation.	MDOT observation was feasible and the approach was satisfactory for the majority of participants.
Krueger *et al*., 2010. [46] **USA**	Videophone utilization as an alternative to direct observation therapy. **International Journal of Tuberculosis and Lung Diseases**	Videophone. Recording was for an average of 20 weeks.	Retrospective study covering period between 2002–2006. 57 patients used videophone technology.	To assess cost effectiveness of the videophone as an option for performing DOT approach in TB patients.	The rate of adherence with TB recorded treatment via videophone was high. A total of US $ 139, 546 was saved on the cost of transport and healthcare provider observation time.	Videophone observation for treatment was a valid method and can be used to improve adherence.
Wade *et al*., 2009. [47] **Australia**	Videophone delivery of Medication Management in Community Nursing **Electronic Journal of Health Informatics**	Video phone. Study was carried out for 6 months.	Pilot study recruited 9 TB patients with mild cognitive/ impairment. Participants aged between 61and 85 years.	To evaluate the usability and potential use of videophone to remotely observe TB therapy administration at the patients’ homes.	Positive attitude recorded toward use of this technology from patients and staff. It is useful for saving time and costs related to home visiting.	Videophone provides an efficient alternative approach to assess therapy at the patient’s home instead of visiting the patient’s home. 6 (67%) patients would like to continue use of the mobile technology.
Smith *et al*., 2007. [48] **USA**	Tele-health home monitoring of solitary persons with mild dementia **American Journal of Alzheimer’s Disease and other Dementias**	Videophone. This study was carried out with patients for an average of 6 months.	Compared video phone monitoring group (8 patients) with a control group (6 patients). Age of participants range (79–85 years).	To assess technical feasibility of the approach, medication administration and mood in patients with dementia.	The technical success of using the videophone was 82%. Video phone adherence was 81%, while adherence was only 66% in control patients.	Telemonitoring of patient treatment via videophone led to maintaining dementia patients’ adherence, but there was no clear influence on patient mood.
Demaio *et al*., 2001. [49]. **USA**	The application of telemedicine technology to observed therapy programme for tuberculosis: A pilot project **Clinical Infectious Disease**	Videophone system was connected with touch phone or television. Study duration was not mentioned.	Pilot study using videophone to connect between patients’ homes and healthcare centre for treatment observation. Six patients used DOT method then switched to using the videophone method.	To assess the use of video phone for direct observation of TB patient medicine administration and compare this with DOT method.	The recorded rate of videophone adherence was 95% while it was 97% for direct observation at the clinic. The average time for video phone observation was 3 minutes, while direct observation at the clinic needed one hour.	Videophone can lead to sustainable adherence and can be a cost effective approach.

DOT = Direct Observation of Therapy

Four retrospective studies reported on the cost effectiveness of MDOT use compared with regular direct observation of patients at the clinic (DOT) [42,44,46,49].

Numerous advantages and several barriers were identified in published studies from a patient perspective and from a healthcare system perspective (Table 2). The majority of the studies revealed that video recording via mobile phone / videophone was straightforward and patients were highly satisfied with using such an approach.

**Table 2:** Benefits and implementation barriers of using MDOT compared with standard DOT at home or in the clinic.

**1. Benefits of using the MDOT approach** **A. For Patients:** ▪ Flexible and convenient ▪ Can be used to improve or maintain treatment adherence ▪ Reduce travel burden for patients who live in rural areas with restricted access to medical staff and resources ▪ Can be used as communication tool between patient and medical staff e.g. information on medication side effects ▪ Can be used for monitoring adherence of immobile and disabled patients ▪ Improves patient privacy and autonomy (e.g. TB patients)
**B. For the healthcare system:** ▪ Reduced number of medical staff needed for observation ▪ Saves staff time on transport to visit patients at home ▪ Cost effective approach compared with traditional DOT ▪ Can be used to observe patients taking medication at the weekend and public holidays ▪ Improves communication between patients and healthcare provider
**2. Impediments to using the MDOT approach** ▪ Requires good mobile telephone network coverage (or availability of broadband internet) ▪ Technical problems (e.g. connectivity with mobile network) ▪ Data security and confidentiality could be compromised when using public telecommunication networks (unless videos are encrypted) ▪ Difficulty in technology use by some patients (e.g. severe arthritis or poor vision)

DOT = Direct Observation of Therapy

MDOT = Mobile Direct Observation of Therapy

Furthermore, most patients believed that the MDOT approach provided a flexible and convenient method of ensuring medication adherence, compared with the inconvenience of attending a clinic [44]. In general, the time taken to record each video was less than five minutes and this did not affect the daily routine of the patients [13,49]. Moreover, healthcare providers were generally satisfied with the MDOT approach [44,45].

Despite the benefits of using the MDOT approach in monitoring medicine adherence at home, some barriers were reported (Table 2). The main impediment to using the MDOT approach at home was poor mobile network or internet coverage. However, ongoing mobile network upgrades and the burgeoning number of patients who have access to broad band internet within their home will help overcome these obstacles. A further barrier was a degree of patient concern regarding confidentiality (specifically mentioned in 4 of the 10 studies). A number of studies highlighted the fact that that they used a protocol to encrypt videos as part of collection and transmission process to reduce the possibility of unauthorised access. A further issue highlighted by Garfein *et al*. [41] was that the MDOT approach may not be suitable for some types of patients, e.g. individuals with severe arthritis or poor vision may be unable to join a MDOT programme unless they have a partner or helper who can assist with the process.

As a supplementary aspect of this RSR of published studies, several ongoing planned clinical trials were identified in the ClinicalTrails.gov website. These clinical studies will evaluate to use the MDOT approach in TB, stoke, sickle cell disease, HIV patients and in opioid dependence (Table 3).

**Table 3:** Ongoing or planned clinical trials using the MDOT approach in assessing treatment administration (information obtained from ClinicalTrial.gov website, accessed on Feb 2016).

Lead researcher	Clinical trial title	Study design	Participants	Intervention	Starting date	Estimated study completion date
Richard Garfeine Department of Medicine, UC San Diego (USA)	Video directly observed therapy (VDOT) for monitoring adherence to latent tuberculosis treatment (LTBI)	Randomised parallel assignment study where video arm compared with attending in-person arm.	Participant age for recruitment: 13 years and older.	Application for smartphone that enables tuberculosis (**TB)** patients to record and send videos of each medication dose ingested. These videos to be viewed by healthcare workers.	January 2016	December 2019
Daniel Labovitz, Montefiore Medical Center (USA)	Using artificial intelligence to measure and optimise adherence in patients on **anticoagulation therapy**.	Randomised intervention; parallel assignment. Compared with unmonitored group.	Participant age for recruitment: 18 years and older.	Mobile App to be downloaded onto a smart- phone. Artificial intelligence and visual recognition technology to be used to verify medication ingestion.	March 2015	March 2016
Susan Creary, Nationwide Children's Hospital (USA)	Electronic hydroxyurea adherence: a strategy to improve hydroxyurea adherence in patients with **sickle cell disease**.	This is a non-randomised intervention. Crossover study design.	Children and adults to be recruited.	MDOT based on patients' smartphones. Electronic reminder alerts sent to patients. Video recording of patients' daily hydroxyurea administration	July 2014	December 2017
Not provided (Moldova)	Virtually observed treatment (VOT) for tuberculosis in Moldova	Randomised parallel assignment compared with control care group.	Participants: age ˃18 years old	**TB** patient treatment to be observed via video submitted via internet instead of in-person attendance. Comparison with normal practice of observing patient at clinic.	October 2015	February 2017
Anthony DeFulio, National Institute on Drug Abuse (NIDA) (USA)	SteadyRx: smartphone ART adherence intervention for drug users	Randomised parallel assignment compared with usual care group	Participants aged 18 to 65 years old.	Mobile App will automatically facilitate consultation of human immunodeficiency virus (**HIV)** patients with care providers, provide reminders when a dose is overdue, and provide electronic remote observation of medication taking.	Not specified	February 2017
Alain Litwin, Montefiore Medical Center (USA)	Using artificial intelligence to monitor medication adherence in opioid replacement therapy	Single group assignment	Participants aged ˃18 years old	This study will use an artificial intelligence platform via mobile app to automatically confirm medication ingestion of opioid dependence. In case of missed dose, an alert message will be sent via text message or Email.	March 2016	March 2017

Furthermore, four conference abstracts were discovered during further manual searches for relevant studies, all of which discussed TB adherence. Three abstracts were preliminary reports of main studies while the fourth [50] reported ≥80% adherence in 34 patients. Finally a search of the internet indicated new developments within the field, e.g. facial recognition in a new Aicure® smartphone app in which video review is carried out automatically [51].

Although there is a scarcity of research on the MDOT approach, the work to date suggests that the approach provides a promising tool to encourage and monitor patient adherence to prescribed medication. As is customary with the RSR methodology, the main findings of the review have been summarised in a short statement for ease of reference. This statement is included in Box 1. As mentioned previously there were no studies which used mobile technology in inhaler assessment.

**Box 1. RSR report—MDOT in treatment adherence**

**Research question**

- What is the current evidence in the published literature concerning the effectiveness of the mobile direct observation therapy (MDOT) approach in treatment adherence?

**The main purpose of this rapid review**

The purpose of the review was to gather evidence about the feasibility and effectiveness of the MDOT approach in monitoring medicine adherence.

**The key messages of this rapid review**

▪ The MDOT approach is feasible and promising for observing treatment remotely
▪ Although several studies have demonstrated the feasibility of recording videos of treatment administration via a mobile device and sharing this with healthcare providers (or real time viewing of treatment administration using a videophone), no robust, randomised studies have been published to date. Several ongoing studies were reported in the ClinicalTrail.gov website in which randomised recruitment is ongoing to larger studies.
▪ The MDOT approach can provide multiple benefits for patients and the healthcare system. The flexible and convenient approach, reduces the burden for patients and saves public resources.
▪ The approach has been used to date mainly in the treatment of TB and Sickle cell disease.
▪ There was a lack of methodological rigor and overall quality in the studies published to date in assessing the effectiveness of MDOT in treatment adherence.
▪ Evidence of the effectiveness of MDOT in assessing and promoting treatment adherence is currently insufficient and, therefore, further research should be conducted in this evolving area.

### Feasibility study–healthy volunteer children

A total of 12 healthy children and 9 parents participated in this study, i.e. three parents had two participating children (Table 4). The mean age of children was 7.1 years (age range 2–12 years). Two thirds of the children were female (n = 8). The majority of participating parents also were female (n = 8). All participants completed recording and submitting videos using the mobile DOT approach for the entire period of the study (14 days).

**Table 4:** Demographics of participating children, MDOT adherence rate and the mobile devices used in the feasibility study.

Patient ID	Parent ID	Child gender	Parent gender	Age of child (years)	No. of uploaded videos	No. of days missed at least 1 video	Mobile DOT adherence %	Device brand used for MDOT
C1	P1	F	F	11	26	1	92.8	ipad (Apple)
C2	P1	F	F	5	25	2	89.2	ipad (Apple)
C3	P3	M	F	7	11	9	39.2	Iphone(Apple)
C4	P4	F	F	5	24	2	85.7	HTC phone (Android)
C5	P5	F	F	6	24	2	85.7	iphone(Apple)
C6	P5	F	F	2	24	2	85.7	Ipad (Apple)
C7	P7	F	F	6	22	3	78.5	Iphone (Apple)
C8	P8	M	F	3	22	3	78.5	Iphone (Apple)
C9	P9	M	M	10	28	0	100	Hudl tablet (Android)
C10	P10	F	F	8	27	1	96.4	Huawei Tablet (Android)
C11	P10	F	F	10	28	0	100	Huawei Tablet (Android)
C12	P12	M	F	12	23	3	89.28	iphone (Apple)

MDOT = Mobile Direct Observation of Therapy

**Fig 2:**
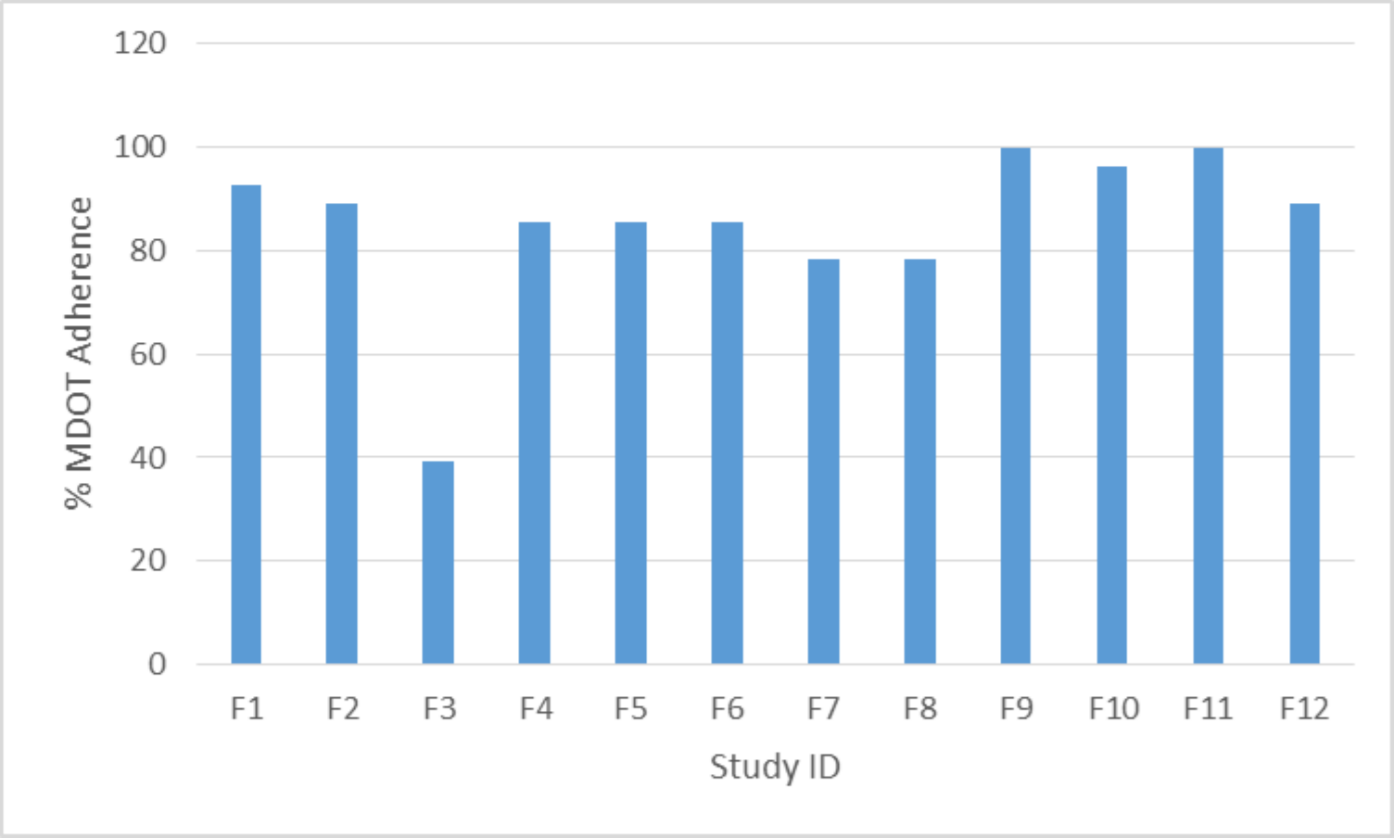
Mobile DOT video upload adherence in the feasibility study.

A total of 284 video clips were uploaded during the study period. The overall adherence rate of submitting videos was 84.5% (Fig 2). Two children (aged > 8 years old) completed the study without missing any video uploads (100% upload adherence; Table 4). Only one participant had an upload adherence rate of less than 75%. The latter was attributed to arrangements at weekends as the child visited a second parent’s home (father) and this parent was not participating in the study. A study which examined adherence to renal medication in children is consistent with our finding that change of routine at weekends can result in a drop in adherence rate [52]. A range of different mobile devices (using either Apple or Android operating systems) were used by the participants in the study (Table 4).

The majority (87.1%) of uploaded videos via the MDOT system were judged to be of acceptable quality, 11.25% marginally acceptable and 1.65% of unacceptable video quality (due to poor lighting at home or improper framing of the video capture).

A total of eight children and nine parents were interviewed after they completed the video upload aspect of the study. The vast majority of children were satisfied with the MDOT system. Parents reported that their children were enthusiastic and interested in the use of the MDOT approach in this project, particularly older children. Older children themselves mentioned that twice daily recoding of their use of the inhaler was fun and enjoyable. In the case of only one child, the parent stated that he got somewhat bored by the end of the two week study. The present findings are consistent with a study carried out in children with sickle cell disease in which children accepted and were satisfied with daily recoding of their medication use, in this latter case over a prolonged period of six months [13]. Participants in the pilot study provided a variety of different suggestions to improve the use of the MDOT methodology, despite most children and parents finding the use of the MDOT system to be very easy and straightforward. The main improvement suggested was to have a more colourful interface and the provision of feedback to the parent/child that the video had been uploaded successfully. Multiple studies have shown that the use of mobile phone apps are acceptable to patients in assisting in the management of HIV, TB and diabetes management [45,53,54] and this was certainly the case in the present study. The training time for each participant on how to capture videos and upload them using the MDOT platform was short, i.e. the session for software installation, MDOT demonstration and training took approximately 30 minutes. With regard to the time required to use the MDOT approach, approximately five minutes was required for the complete MDOT process, including preparation time. These times are consistent with other MDOT studies that reported time requirements of less than 5 minutes [13,42,49].

In general, there were no major technical problems with the current MDOT system during the study. One parent living in a rural location reported some difficulty with a slow internet connection. Another parent had some difficulty with the size of the video clips, however, this was easily overcome by downgrading of the resolution in the mobile phone camera. It was observed, based on the videos submitted, that poor lighting in the room used to prepare videos or the participant being too far away from the mobile phone affected video clarity. This point was also highlighted by Hoffman *et al*. [45]. It was noted that the mobile device stand provided to each participant helped participants in the self-capture of the video clips without any assistance from others. In addition, it enabled participants to control the framing themselves and facilitated video stability. One parent reported the benefit of the mobile stand, as follows: “Not at all complicated. We had to adjust the little device for propping up the iPad with. She knew when the angle is correct. She is pretty good checking it. She will be keeping an eye to see that it was at the right angle” (parent of child aged 11 years). The results of this feasibility study confirm that this monitoring system for inhaler use is feasible and could be suitable to monitor child inhaler use within a clinical setting. Furthermore, it has the potential to monitor the correct steps of inhaler use and in monitoring adherence in children.

### Pilot study in children with asthma

**Fig 3:**
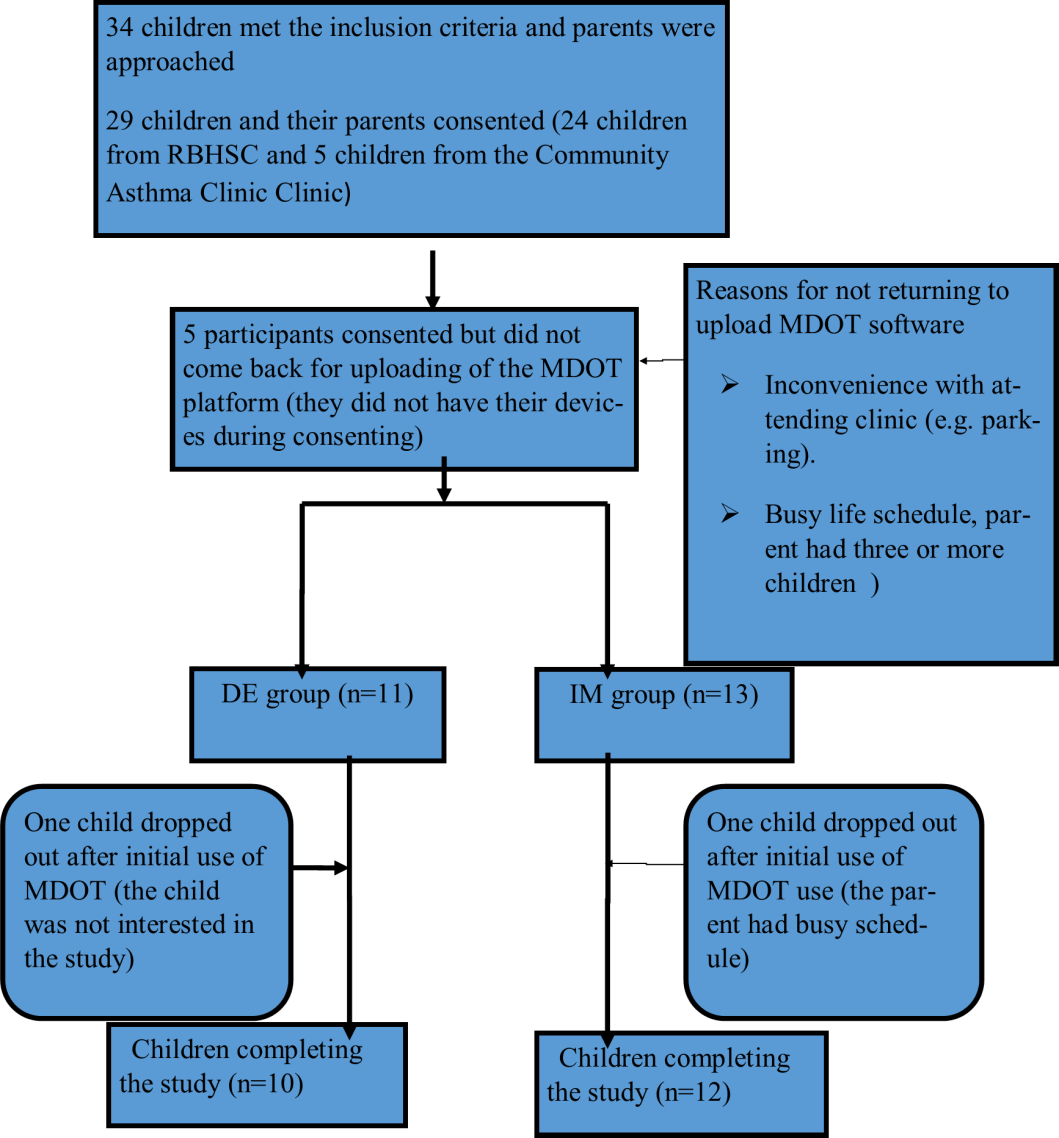
Number of children who participated in the pilot study in children with asthma.

A total of 34 eligible patients were approached across the two study sites. Twenty two children and their parents completed the study (Fig 3). Reasons for non-participation and withdrawals from the study are reported within Fig 3. For the purpose of this pilot study analysis, due to the small numbers of patients involved, data sets from the IM and DE groups were combined and data were compared between baseline and 12 weeks post the initiation of DOT.

#### Patient demographics

The gender distribution of children was 17 male and 5 female. The age range of children entering the study was 2–16 (mean 9.4 + 3.7 years). The mean (+ SD) ACT scores in the participating patients were 13.1+ 5.7 at baseline while the mean (+ SD) FeNO levels at baseline were 38.7 + 28.8 PPM. Over 50% of the cohort (12/22) had been prescribed oral steroids in the year prior to study entry and approximately 40% (9/22) had used ‘out of hours’ GP services in the previous year. The majority (13/22) had a comorbid illness, primarily hay fever and eczema.

#### Inhaler technique

Despite all children being able to demonstrate good inhaler technique during study enrolment at the clinic, a total of 77% (17/22) of the participants had partially effective or poor inhaler technique in the first week of MDOT assessment. This is an important finding indicating that satisfactory technique at the clinic is not carried forward to home use. By week 4 of MDOT engagement, approximately 90% (17/19) of the children had inhaler technique which was judged as effective (Table 5). At this stage, 3 patients had stopped recording their inhaler use and were not responding to phone calls or text messages. By week 5 all children still enrolled in the study (n = 18) were judged to have effective inhaler technique, i.e. inhaler technique improved after tailored inhalation instructions over the telephone by a member of clinical team. From inhaler observation, slow inspiration was found to be the most common mistake related to the use of dry power inhalers while forgetting to shake the inhaler before use and poor sealing of lips around the mouthpiece were common with pressured aerosol inhalers.

**Table 5:** Classification of inhaler technique in children during the 6 week use of the MDOT approach in the pilot study in children with asthma.

Child ID	1st week	2nd week	3rd week	4th week	5th week	6th week
DE1	P. effective	Effective	Effective	P. effective	Effective	Effective
DE2	Poor	P. effective	Effective	Effective	Effective	Effective
DE3*	P. effective	P. effective	Effective	Effective	----------	-----------
DE4	Effective	Effective	Effective	Effective	Effective	Effective
DE5	P. effective	Effective	P. effective	Effective	Effective	Effective
DE6	Poor	P. effective	P. effective	Effective	Effective	Effective
DE7	Effective	Effective	Effective	Effective	Effective	Effective
DE8	Poor	Effective	Effective	Effective	Effective	Effective
DE9	Effective	Effective	Effective	Effective	Effective	Effective
DE10*	P. effective	P. effective	------------	------------	----------	-----------
IM1	P. effective	Effective	P. effective	Effective	Effective	Effective
IM2	P. effective	Effective	Effective	Effective	Effective	Effective
IM3	P. effective	P. effective	Effective	Effective	Effective	Effective
IM4	P. effective	P. effective	Effective	P. effective	Effective	Effective
IM5	Effective	P. effective	Effective	Effective	Effective	Effective
IM6*	Poor	------------	-----------	-----------	----------	----------
IM7	P. effective	P. effective	P. effective	Effective	Effective	Effective
Im8	P. effective	P. effective	Effective	Effective	Effective	Effective
IM9	P. effective	P. effective	Effective	Effective	Effective	Effective
IM10	P. effective	Effective	Effective	Effective	Effective	Effective
IM11	P. effective	Effective	P. effective	Effective	Effective	Effective
IM12*	Effective	Effective	Effective	-------------	----------	-----------

* Stopped recording and did not respond to phone or text messages

P. effective = partially effective technique

MDOT = Mobile Direct Observation of Therapy

#### Influence of MDOT on outcomes

A summary of the data on impact of the MDOT intervention on asthma outcomes, 12 weeks post the initiation of the intervention is presented in Table 6. The two main markers of asthma control (ACT and FeNO) were improved significantly (P<0.05) at the 12 week assessment; the minimum clinical difference required (i.e. 3 points in the ACT) to mark improved asthma control was achieved [55]. The MDOT approach also led to a reduction in the mean FeNO value (PPM) to normal levels, i.e. 38.7+28.8 (n = 22) to 19.3 + 14.4 (n = 16) (Table 6). Within this small pilot sample spirometry values did not vary to a statistically significant degree between baseline and 12 weeks (P>0.05).

**Table 6:** Summary data on impact of MDOT intervention on asthma outcomes (at 12 weeks post initiation of MDOT) in the pilot study in children with asthma.

Outcomes	Baseline Mean ± SD	12 weeks post MDOT Mean ± SD	p–value
Asthma control ACT	13.1±5.7	17.8±4.3	0.007*
FeNO (PPM)	38.7±28.8	19.3±14.4	0.019*
Spirometric value FEV_1_%	91.9±13.1	92.8 ± 10.5	0.721
FEV_1_/FVC%	92.6± 10.9	92.8±10.5	0.952
Child QOL (PAQLQ) (≥9 years)	3.8±1.6	4.9±1.5	0.079
Parent QOL (PACQLQ)	3.7±1.4	5.4±1.3	0.001*
Clinical assessment of asthma Controlled	0	7	-
Partly controlled	8	11	-
Uncontrolled	14	4	-

^*^ Independent T test (Significant at p<0.05)

MDOT = Mobile Direct Observation of Therapy

All parents/guardians (n = 22) completed the QOL questionnaires (PACQLQ) at baseline while 19 parents completed the questionnaire at 12 weeks post initiation of DOT. The results demonstrated improved QOL at 12 weeks compared with baseline (P< 0.001). A mean score of > 4 (moderate to excellent QOL) was achieved by participants at week 12. A total of 12 children > 9 years old completed the QOL questionnaire (PAQLQ) at baseline and at 12 weeks post initiation of MDOT. An improvement in the mean score of greater than one unit was achieved, however, this did not reach statistical significance (p = 0.079; Table 6). A statistically significant correlation were found between ACT scores and parent QOL scores at baseline (r = 0.55; p<0.001) and at 12 weeks post MDOT initiation (r = 0.74; p<0.001). Similarly, statistically significant correlations were found between ACT scores and child QOL scores at baseline (r = 0.75; p<0.001), and at 12 weeks post MDOT initiation (r = 0.65; p<0.001). Clinician assessment of asthma control indicated that at 12 weeks post initiation of the intervention, patients were moving towards the partially controlled or controlled categories, with a marked reduction in the patients deemed uncontrolled i.e. 14 at baseline vs 4 at 12 weeks (Table 6).

#### Adherence to video upload

A total of 1083 video clips were recorded and uploaded during the six week MDOT period by participants. Using the video upload approach, 72.7% of the children (16/22) achieved moderate adherence to video uploads (≥ 50%) for the intervention period. The frequency / reasons for not uploading videos were as follows: busy schedule (e.g. school exam)– 6; child visits father’s home at weekend and father not trained in MDOT process– 3; forget to take video– 3; large video file size -3 (corrected by decreasing video resolution on mobile phone); lost phone– 2; no space on mobile phone storage– 2; child was unwell (e.g. gastroenteritis)– 2; mother was admitted to hospital– 2; travelling outside the country– 1. A total of four participants had a video upload adherence rate of less than 30%. There is clear scope for significant improvement in this aspect of MDOT including automated reminders, video compression (to reduce memory requirements on the mobile device), familiarisation of more than one family member with MDOT and perhaps a reduced frequency of monitoring (e.g. one upload each evening, with a tick box to confirm whether the inhaler has been used in the morning).

#### Adherence to inhaler use (GP records and MARS)

All of the GP prescribing data requested were received. Using the cut point of 80%, 10 children (45.4%) were deemed adherent and 12 children (54.6%) were classified as non-adherent in the 12 months prior to enrolment in the present study.

All 22 parents/guardians of the children who participated in the study completed the MARS questionnaires at baseline. Using a cut point of ≥ 80% of the maximum score achievable to represent adherence, the level of non-adherence reported for the participants was 18.2% at baseline. Nineteen parents completed the questionnaire at 12 weeks with 0% non-adherence reported. Regarding the child MARS, 12 children (≥ 9 years) completed the questionnaire at baseline with a reported non-adherence rate of 61.5%. By 12 weeks post MDOT initiation, again 100% adherence was reported by those children. Although the numbers within the pilot are small, the strong trend towards improvement in self-reported adherence is clear.

#### Parent feedback on use of MDOT

Feedback from parents was very positive. They found the MDOT approach: ‘Very easy to access with patient ID, take a video and simply upload’. There was general support for loading videos twice per day e.g. ‘I think loading videos twice daily was worthwhile as it drew emphasis to the need to take medication’. Another parent commented ‘It seems normal to do it twice because it only lasts for a few minutes and does not take up any time’. Parents were asked whether 6 weeks was an appropriate duration for use of the MDOT intervention. The responses here were generally supportive of a 6 week intervention, however, some parents thought the intervention could be longer or indeed shorter. A period of 4–6 weeks seems ideal. Variable answers were provided to the question regarding what age a child could use the MDOT independently. The most pertinent answer was that: ‘If a child is able to use a smartphone, they would not find any problems’ and indeed one parent reported: ‘My child of 5 years old was able to use it’. Other parents suggested ages of 8–13 years old as being appropriate for self-use of MDOT.

The main barrier reported by parents was slow uploading of videos when internet / mobile network speeds were slow and on a few occasions videos did not upload. Parents were asked to suggest improvements to the MDOT platform. One suggested: ‘Maybe a little alarm would be useful’ while another suggested that there ‘could be a place to upload comments along with the video of inhaler use to help the doctor know how the child is getting on’. Both those suggestions are being incorporated into a redeveloped MDOT platform (www.continga.co.uk).

Advantages of using the MDOT proffered by parents were focused on (i) observing inhaler technique, (ii) monitoring / improving treatment and (iii) working as a reminder. Typical comments within these three areas respectively were: (i) ‘I think it is a great idea because the doctor is able to see how the child is using the inhaler’; ‘It has been a great help in finding out faults’, (ii) ‘I think it is great as [it] showed children are getting [the] correct treatment’; ‘I think 6 weeks was ideal because it got my son into a routine and he still is on it’; ‘It is good to learn how to control your child’s asthma’; ‘It gives parent peace of mind to know that the child with asthma is controlled right’, (iii) ‘It helped my son remember to take his inhalers twice daily’. Our findings support the views of other users (with other chronic illness) that the MDOT approach is convenient, not time consuming and with minimal disruption can be integrated into routine daily activities (Table 1).

As detailed earlier a small number of parents agreed to use the MDOT but either did not get started with the MDOT monitoring or discontinued early. Reasons for this were generally related to practical matters e.g. parking difficulties at hospital (parent did not have mobile phone at clinic visit and did not return to get the MDOT software uploaded; not being at home when child was using inhaler due to shift work; difficulty getting up in the mornings to help child. Approaches to overcome some of these logistical challenges are currently being developed by the research team.

The overall assessment of the pilot study by the researchers is that all aspects of study ran very much as planned. Although this is a small pilot study, the clinical outcome improvements at 12 weeks post MDOT initiation were encouraging as was the acceptability of the MDOT approach to parents and children. The focus of the pilot study was on improvements from baseline when the MDOT intervention was in place. It was noted, however, that there were improvements in the DE group outcome measures (particularly FeNO) at the end of the 6 week period at the start of the study i.e. before the MDOT intervention was initiated in that group. This was likely due to their enrolment in a clinical trial and becoming better engaged in their therapy because of this (Hawthorne effect). These aspects will be explored further in a larger study that is being planned by members of the research team. The MDOT approach has been used successfully for prolonged periods in TB management. However, since medication adherence is known to drop off over time in children [56] future research is required to ascertain if adherence to MDOT deteriorates over longer term use in inhaler management. Further work is also required to ascertain exactly how long MDOT is needed for to give rise to sustained improvement in inhaler technique and if further specific interventions are required to give rise to sustained adherence to the MDOT intervention.

## Conclusions

MDOT is a technology that has the potential to be a cost effective approach (patient does not have to attend clinic or observer does not have to visit patient) to direct observation of therapy administration, the latter being one of the most accurate methods of evaluating adherence. It could also be potentially useful for children whose caregivers have busy schedules and find it difficult to attend scheduled clinics. Use to date, as confirmed by the rapid systematic review, has been limited mainly to TB and sickle cell disease and there have been no published reports on the use of MDOT to monitor inhaled therapy. Due to the increasing incidence of childhood asthma worldwide, there is a need for new innovative approaches to support children and their parents with asthma management, especially since national and international guidelines have advised healthcare providers to periodically assess inhaler use as part of asthma management [57,58].

After successful testing of the feasibility of using MDOT to assess inhaler technique and adherence in healthy volunteer children, the present programme of work went on to assess, in a pilot study, the impact of the approach in children with poorly controlled asthma. The results from the pilot study were also very encouraging, with children who received the MDOT intervention showing significant improvement in the two main markers of successful asthma management, i.e. the ACT and FeNO. It is perhaps worthwhile restating that all patients enrolled were receiving care from a specialist asthma clinic and had received best practice education ahead of joining the study.

Checking the uploaded videos allowed patients to be contacted if their inhaler technique was deteriorating (which it often did during the early phase of the intervention) or if videos had not been uploaded. This approach is obviously much more cost efficient when compared with DOT. Although it can be used to monitor inhaler adherence, a potential issue is that a patient may use the inhaler but forget or not bother to make and upload the video of the administration. This aspect of the overall intervention requires some further thought, perhaps an inbuilt reward system to encourage full engagement would be helpful. Also it could be the case that once per day uploading of inhaler use would be sufficient to engage patients / parents and check on inhaler use, with a simple tick box to be completed to record the second daily use of the inhaler. It was noted in the feasibility and pilot studies that the MDOT approach and the IT platform employed was easy to use and with the exception of a few instances where internet connectivity was poor, good quality videos (including the sound of inhaler activation and patient inspiration) could be easily captured and transmitted. It was also noted that the approach allowed the development of a bilateral partnership and communication between the treatment team and the parent / guardian of the child or indeed older children themselves.

This is the first research study which has examined adherence technique and inhaler use in children using the MDOT approach. The approach could be particularly useful in ensuring asthma patients are adherent to low cost standard therapies ahead of being prescribed expensive biological treatments. It also has the economic advantage that adherence can be monitored A major advantage of the approach is that individual steps in inhaler use can be easily followed, regardless of inhaler type. The very positive results should promote further adoption of, and research on, this approach in children with asthma and in other conditions across the wider population in an attempt to overcome the scourge of non-adherence which has plagued pharmacotherapy over many years.

## Supporting information

S1 PRISMA Checklist PRISMA 2009 checklist MDOT.doc. (DOC)

S1 CONSORT Checklist CONSORT 2010 Checklist 300817.doc. (DOC)

S1 Flow PRISMA 2009 flow diagram MDOT.doc. (DOC)

S1 Protocol RESEARCH PROTOCOL.docx. (DOCX)
