# Supplementary material for: Mobile direct observation of therapy (MDOT) - A rapid systematic review and pilot study in children with asthma
Source: PLoS One. 2018 Feb 5;13(2):e0190031. doi: 10.1371/journal.pone.0190031 (PMC5798760; doi:10.1371/journal.pone.0190031)
Supplement: S1 Flow — (DOC) [file pone.0190031.s003.doc]

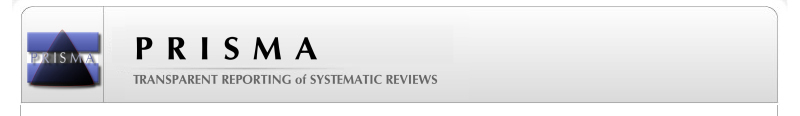
**PRISMA 2009 Flow Diagram**

**Screening**

**Included**

**Eligibility**

**Identification**

Records identified through database searching
(n = 754 )

Additional records identified through other sources
(n = 5 )

Records after duplicates removed
(n = 320 )

Records screened
(n = 320 )

Records excluded
(n = 306 )

Full-text articles assessed for eligibility
(n = 14 )

Full-text articles excluded, with reasons
(n = 4 )

Studies included in qualitative synthesis
(n = 10 )

Studies included in quantitative synthesis (meta-analysis)
(n = )
