# Supplementary material for: Mobile direct observation of therapy (MDOT) - A rapid systematic review and pilot study in children with asthma
Source: PLoS One. 2018 Feb 5;13(2):e0190031. doi: 10.1371/journal.pone.0190031 (PMC5798760; doi:10.1371/journal.pone.0190031)
Supplement: S1 Protocol — (DOCX) [file pone.0190031.s004.docx]

**RESEARCH PROTOCOL**

**Evaluation of a mobile direct observation therapy (DOT) approach in children and young people with asthma**

**Pilot study**

**^1^Fahad ALQahtani, James C. McElnay^1^, Michael P. Rivey^1^ and Michael Shields^2^**

**^1^Clinical and Practice Research Group, School of Pharmacy, Queen’s University Belfast.**

**^2^Centre for Infection and Immunity**, **School of Medicine, Dentistry and Biomedical Sciences, Queen’s University Belfast.**

**Background**

Asthma is the most common chronic disease in childhood. It is defined as a chronic inflammatory disorder of the airways in which many cells and cellular elements promote airway obstruction and hyper-responsiveness (GINA, 2012). According to the World Health Organization (WHO, 2014), asthma is estimated to affect approximately 253 million people worldwide. Despite advances in biological and pathological research, the prevalence of asthma in children has significantly increased over the past decade (Massingham *et al*., 2014).

Moreover, the economic burden of asthma is increasing. In the US, it is estimated that the yearly cost of asthma in children and adults is around $1.48 billion (Price *et al*., 2013). A decade ago, the total annual asthma expenditure in the UK was determined to be £752.6 million with 8% of costs associated with hospital admission, 13% attributable to general practitioner consultations and 79% due to prescription costs (Gupta *et al.*, 2004).

The British Thoracic Society (BTS/SIGN, 2012) and Global Initiative for Asthma (GINA, 2012) guidelines provide the background definitions for three broad categories of asthma control. They include **controlled** (no nocturnal wakening, infrequent short acting beta 2 agonist (SABA) use e.g. < 2 puffs/week, occasional mild symptoms e.g. with exercise and no exacerbations in last 3 months), **partially controlled** (nocturnal wakening< 3 nights/week, SABA use e.g. < 4 puffs/day, mild limitation in exercise tolerance due to asthma, and 2 or fewer mild exacerbations in the previous 3 months) or **uncontrolled** (nocturnal wakening 4-7 nights/week, SABA use e.g. > 5 puffs/day, limitation in exercise tolerance due to asthma and or significant asthma exacerbations requiring oral steroid, Emergency Department attendance or hospital admission in the previous 3 months). Children who present with partially controlled or uncontrolled asthma can be divided into difficult to treat asthma (DTA) and true severe therapy resistant asthma (STRA) after careful investigation (Hedlin *et al.*, 2012). DTA occurs when asthma is uncontrolled but the impact of concomitant disorders and the basics of asthma care (inhaler technique and adherence) have not been adequately resolved.

Many children with asthma can achieve symptom and disease control by using inhaled corticosteroid (ICS) therapy combined with a long acting B_2_ agonist (LABA) and/or a leukotriene receptor antagonist(LTRA) (International ERS/ATS guideline, 2014). However, a number of children with asthma experience frequent symptoms despite being prescribed high dose ICS (Nagakumar and Thomas, 2013; Hedlin *et al.*, 2014)**.**

Drug delivery by inhalation of various medications is the most common treatment approach for asthma in all patient populations. Inhalation therapy offers rapid onset and improved efficacy compared to systemic drug delivery (Bisgaard, 1997).While ICS therapy is well accepted as the foundation of optimal therapy for most asthma patients, efficacy of the therapy depends on drug being delivered correctly into the lungs and taken on a regular basis as a preventer therapy(Machira *et al*., 2011).

It has been suggested that good inhaler technique is an essential aspect of optimal asthma management. Education on inhaler technique has been shown to improve self- management and adherence to asthma therapy. Evidence is available demonstrating that asthma outcomes correlate with proper inhaler technique and that periodically assessing this treatment aspect has a positive impact on asthma control(Bryant *et al*.,2013; AL-Jahdali *et al*., 2013). Moreover, early and repeated tailored education and follow-up assessment can result in sustained good inhaler technique (Klot *et al*., 2011).However, many asthma clinics treating children are unable to effectively evaluate these important patient treatment variables.

Adherence to pharmacotherapy is also considered an important basic tenet of asthma management. Adherence can simply be defined as the extent to which a patient follows the instructions of the prescriber (Osterberg and Blaschke, 2005).While inhaled therapy technique is an essential feature of asthma pharmacotherapy, some patients with good technique may have poor inhaler adherence. Available data suggest the average medication adherence to ICS in children is only approximately 50% (Fish *et al*., 2001; Osterberg and Blaschke, 2005).

It is known that adherence to ICS in children is a complicated matter influenced by diverse issues including socioeconomic factors, parents’ beliefs about asthma and asthma medications, and poor patient-doctor communication (Osterberg and Blaschke, 2005; Armstrong *et al*., 2014). In addition, parent/guardian health issues such as depression or substance abuse may negatively affect adherence in young children as a result of the parent/guardian’s failure to remind, or assist the child in the use of their medication (Brackis-Cott *et al.*, 2003). Non-adherence to medication can have various negative patient consequences such as more frequent clinic visits, disease exacerbations, hospital admissions, and increased cost of care (AL-Jahdali *et al*., 2013).Indeed, a recent review of childhood asthma deaths in SE England showed that medication non-adherence was a contributing factor in more than 50% of cases, including patients with milder disease (Anagnostou *et al*., 2012)

Despite being an area of active research, many interventions such as isolated educational efforts are ineffective in improving adherence (Drotar and Bonner, 2009).In a qualitative study in the primary care setting, asthmatic children of parents who expressed medication beliefs of a high concern–low need pattern had poor outcomes. Researchers subsequently showed that these beliefs could be modified by repeated tailored education and close follow-up, to result in high medication adherence. They concluded that “it is not the education per se, but rather the intensity, quality and frequency of education about self-management and follow-up that help to improve adherence” (Klot *et al.*, 2011).A multicentre clinical trial in the USA was designed to determine what medication was best to add in children with severe asthma already taking ICS and LABA. Interestingly, the trial was cut short due to an inability to recruit an adequate number of children. Patient improvement while under close supervision during the run in period was the main reason for the lack of patient recruitment. (Strunk *et al.*, 2008)

Different methods used to assess patient adherence to prescribed therapy have resulted in variable outcomes. Adherence can be measured by collecting data from patients or their parent/guardians, pharmacy dispensing records, electronic monitoring devices, or patient observation (Osterberg and Blaschke, 2005).Sincethe approaches have produced variable results, it can be concluded that presently there is no preferred method to effectively measure or improve medication adherence in asthma (Sumino and Cabana, 2013).

Direct observation therapy (DOT) is recommended by the WHO to promote adherence in the management of tuberculosis (TB) and this approach was adopted in 187 countries by 2005 (WHO, 2010). DOT allows assessment of correctly taking and completing therapy, but also can be valuable to promote adherence to medication use. DOT involves a healthcare professional observing patients taking their medication at home or in the clinic (Dosumu, 2001; Otu, 2013).The DOT approach to TB management has been utilised successfully in different countries as this approach has been shown to enhance medication adherence, improve outcomes including microbiologic success, and decrease acquired drug resistance to therapy (Favorov *et al.*, 2012; Walley *et al*., 2001; Pasipanodya and Gumbo, 2013).

While data exist to show that observation of inhaler technique can positively affect asthma management, there are no published studies using a DOT approach as a means to improve medication adherence. However, it has been suggested that using DOT with nurses directly observing asthmatic children’s inhaler technique and adherence, for example at school or home, would be very resource intensive and likely not feasible for continued use (Otu, 2013).

In recent years, there has been widespread use of mobile smartphone and computer devices within the healthcare environment. A recent survey of teenagers and caregivers in urban paediatric practices showed that 84% of respondents were smartphone owners, a finding that was independent of age group, gender, ethnicity, and socioeconomic status (Singh *et al.*, 2014).DeMaio *et al.* (2001) conducted a pilot study that compared videophone telemedicine (V-DOT) to standard DOT (S-DOT) in the treatment of TB. In the videophone group, patients took videos of medication administration that were provided to clinicians involved in their management. The adherence rate was 95% for V-DOT compared to 97.5 % for S-DOT, but personnel time was decreased by 288 hours with V-DOT in only 6 patients. Similarly, a telehealth videophone home monitoring approach to TB was shown to enhance treatment compliance and use less clinic resources in a larger study by Wade *et al.* (2012).

More recently, Creary *et al.* (2014) developed a novel mobile DOT approach utilising smartphone and computer devices that resulted in a median monthly observed adherence of 93.3% over the 6 month trial in children with sickle cell disease. Finally, specific to asthma patients, Vasbinder *et al*., (2013) has developed a study to access the effectiveness of using mobile phone text-messages to remind and encourage non-adherent patients to take their medication and thereby improve control of their disease; results of the study are not yet available. Limited available evidence, therefore, suggests that communication technology could be utilised to achieve therapeutic goals of correct administration technique and improved adherence to therapy in asthma patients while limiting resource utilisation.

**Aims and objectives**

The principal aim of this study is to evaluate the feasibility and clinical impact of a mobile DOT approach (via video capture in the patient’s home) on the administration technique for, and adherence to, ICS in children with partially controlled or uncontrolled asthma. A secondary aim is to determine factors that influence adherence to ICS in children with asthma.

The specific objectives are to:

1. Investigate the feasibility, practicality, and persistence of a mobile DOT technology approach in the home setting to assist with disease management of children and young people with partially controlled or uncontrolled asthma.
2. Assess inhaler technique and adherence to ICS therapy by use of DOT videos, parent/guardian and child self-report questionnaires (Medication Adherence Report Scale (MARS)), pharmacy records and patient’s general practitioner (GP) records.
3. Evaluate the impact of the DOT intervention on asthma clinical outcomes including physician assessment of disease control, medication changes, asthma control tests, health-related quality of life and pulmonary function studies in participating children.
4. Identify parental/guardian and patient factors that influence adherence to ICS in participating children and young people with asthma

**Study design**

A randomised intervention trial will be used. Participants will be randomised to either an immediate (I-med) or a delayed (D-med) mobile technology DOT intervention. Randomisation will be restricted based on two factors including age (categorized as Young Children ages 2 up to 5 years, Children ages 5 up to 12 years and Young People ages 12 up to and including 16 years) and patient gender (Gore, 1981).

Both subject groups will receive standard-of-practice asthma education and management in the Asthma Clinic prior to study enrolment. Participants allocated to the I-med group will take part in the mobile technology DOT intervention for the first 6 weeks. Outcomes will be evaluated at the start (week 0) and end of the 6 week intervention period and during clinic visits at weeks 12 and 18 for follow-up. Those participants allocated to the D-med group will have the DOT intervention started after a 6 week “intervention-free” interval with usual Asthma Clinic care. Outcomes in the D-med group will be assessed at baseline (week 0), week 6 (intervention start), week 12 (end of intervention), and at weeks 18 and 24 for follow-up. (see Figure 1)

**(a) Study site**

Parents/Guardians and their children will be recruited during outpatient appointments at the Belfast Health & Social Care Trust (Royal Belfast Hospital for Sick Children; Community Asthma Clinic).

**(b) Recruitment of participants**

The parents/guardians of children and young people with asthma (aged from2 to16 years) who have continuing symptoms of partially controlled or uncontrolled asthma despite being prescribed ICS therapy will be invited to participate(Invitation letter; Appendix 1). Descriptive information about the study will be provided to both the parent/guardian (Parent/Guardian Information Sheet; Appendix 2) and their children if over the age of 6 years, using age appropriate study information sheets (Appendices 3 and 4). Children will only be included in the study after obtaining written informed consent from their parents/guardians (Appendix 5) and assent from children older than 6 years (Appendix 6). The ability of children to give assent will be determined by their hospital doctor.

**(c) Sample size calculation**

The variability in quality of life (QOL) score changes in a similar population was reported in a pilot study to be 1.3 units (Young *et al.*, 2001). To have an 80% power to detect a statistically significant (P<0.05; two-tailed Student t-test) difference for effect of the intervention between groups of 1 QOL unit would require 36 children per group.

**(d) Inclusion/Exclusion criteria**

Inclusion criteria:

1. Children and young people aged from 2-16 years with apparent partially controlled or uncontrolled DTA. The children will have asthma symptoms despite being prescribed ICS (> 400 mcg/day for children < 5 years, 800 mcg/day for children > 5 years) and a second line therapy such as a LABA, LTRA, or theophylline (Nagakumar and Thomas, 2013).
2. One member of the household has access to a smartphone, tablet or other mobile device that is capable submitting a video image to an internet accessible repository. This person must have access to the device for the entire period of the intervention phase (6 weeks) of the study.

Exclusion criteria:

1. Children whose asthma symptoms are controlled.
2. Children and/or parents without access or unwilling to allow use of a suitable mobile device for the study.

Figure 1. Randomized intervention trial of mobile DOT approach in children and young people with uncontrolled asthma.*

**DOT intervention trial ***

Immediate group n=36 *asthma education

Delayed group n=36 *asthma education

Week 0 (baseline)

DOT intervention – free for 6 weeks

Start of DOT intervention for 6 weeks

Assessment: Start DOT intervention for 6 weeks

Assessment: stop DOT intervention

week 6

Assessment: stop DOT intervention

Follow- up assessment

week 12

Follow- up assessment

Follow- up assessment

week 18

Follow- up assessment

week 24

* all participants will have received standard asthma education currently used within the Trust before randomisation

**Intervention**

**Intervention and data collection**

The project will utilise a simple application (app)set up on each participant’s or parent/guardian’s mobile smartphone, tablet, or other device that will allow video capture and transmission of ICS inhaler administration. Before the study intervention period is initiated in a given subject, the participant and/or parents/guardians will be trained in use of the video DOT application and how to upload the videos to a web-based repository. As a general rule, it would be expected that young people greater than 12 years of age will record and upload the video themselves, while parent/guardian assistance will be needed for younger children. All mobile devices will be tested after installation to make sure the Dropbox®([https://www.**dropbox**.com](https://www.dropbox.com))repository connection is active and ready to use.

Usual asthma care for the participant will be adopted throughout the study. Regarding the inhaler use, children aged 5 years or older may administer the medication themselves while a parent/guardian will be responsible for inhaler administration in patients 2 up to 5 years of age. However, parent/guardian administration will be allowed if that is usual asthma care in any age child.

Patients who are enrolled in the study will be randomised into two groups (I-med or D-med) as previously described. Participants or parents/guardians of participants will be asked to capture a DOT video twice daily (morning and evening) on their mobile devices of ICS use by the patient. It will be requested that morning videos occur before 9am on school days and before 11am on weekends and holidays, and evening videos occur between the evening meal and bedtime. In the I-med group, participants will be asked to upload daily video recordings via the internet to a repository for a 6 week period at the start of the study. Participants in the D-med group will be free from DOT for the first 6 weeks of the study and will be asked to carry out their usual therapy management. After that, DOT as described above will be applied for the next 6 weeks.

Researchers will evaluate the DOT uploads daily through their password protected access to the video repository. Children and their parent/guardians also will have access to their own videos within the password protected repository. A research team member will follow up with parents/guardians and/or a participant by telephone if two consecutive days of video uploads are missed. The asthma clinical team (consultant/nurse) providing the usual asthma clinical management will be notified about children identified with poor inhaler technique.

**Outcome measurements**

**Baseline (at study initiation)**

- Participant demographic (age, gender, postcode, household smoking status) and relevant clinical data including laboratory data (e.g. total & specific IgE) and comorbid conditions. (Appendix 7)
- Clinician assessment of asthma severity and degree of disease control, i.e. controlled, partially controlled, or uncontrolled.
- Asthma medication profile and any changes made at clinic visit
- Number of asthma ‘attacks’, oral corticosteroids courses, and emergency attendances over the previous 6 weeks.
- Spirometry measurement; fraction of exhaled nitric oxide (FeNO)
- Self-reported Medication Adherence Report Scale (MARS) for parent/guardian and child if 9 years of age or greater(Appendices 10 & 11) (Horne *et al.,* 1999; Horne and Hankins, 2008)
- Interview-administered Beliefs about Medicines Questionnaire (BMQ) for parent/guardian and child9 years of age or greater (Appendices 12 & 13) (Horne *et al.,* 1999)
- Interview-administered Paediatric Asthma Quality of Life Questionnaire (PAQOLQ)if 9 years of age or greater, or the Paediatric Asthma Caregiver Quality of Life Questionnaire (PACQOLQ) (Appendices 14 & 15) (Juniper *et al.,* 1996)
- Interview-administered Asthma Control Test (ACT) or Childhood Asthma Control Test (C-ACT) (Appendices 16 & 17) (Nathan *et al.*, 2004; Liu *et al.*, 2007)
- Self-reported Centre of Epidemiological Studies Depression Scale (CES-D) completed by parent/guardian. Total scores range from 0 to 60 with a score > 16 representing depressed mood (Appendix 18) (Radloff 1977). A CES-D score indicating depressed mood in the parent/guardian will be reported to their GP.
- ICS inhaler technique

**At 6 weeks**

- Clinician assessment of asthma control
- Asthma medication profile and any changes made at clinic visit
- Number of asthma ‘attacks’, oral corticosteroids courses, and emergency attendances since baseline visit.
- Spirometry measurement; FeNO.
- Self-reported MARS
- PAQOLQ/PACQOLQ and ACT/C-ACT
- ICS inhaler technique

**At 12 weeks**

- Clinician assessment of asthma control
- Asthma medication profile and any changes made at clinic visit
- Number of asthma ‘attacks’, oral corticosteroids courses, and emergency attendances since baseline visit.
- Spirometry measurement; FeNO.
- Self-reported MARS
- PAQOLQ/PACQOLQ and ACT/C-ACT
- ICS inhaler technique

**At 18 weeks**

- Clinician assessment of asthma control
- Asthma medication profile and any changes made at clinic visit
- Number of asthma ‘attacks’, oral corticosteroids courses, and emergency attendances since baseline visit.
- Spirometry measurement; FeNO.
- Self-reported MARS
- PAQOLQ/PACQOLQ and ACT/C-ACT
- ICS inhaler technique

**At 24 weeks (D-med group only)**

- Clinician assessment of asthma control
- Asthma medication profile and any changes made at clinic visit
- Number of asthma ‘attacks’, oral corticosteroids courses, and emergency attendances since baseline visit.
- Spirometry measurement; FeNO.
- Self-reported MARS
- PAQOLQ/PACQOLQ and ACT/C-ACT
- ICS inhaler technique

**At study end (both groups)**

- GP prescribing records for the 12 months leading up to, and during the study time period
- Community pharmacy dispensing records for the 12 months leading up to, and during the study time period

Clinician assessment of participant asthma severity will be defined according to accepted guidelines and asthma control will be categorized as uncontrolled, partially controlled, or controlled (BMT/SIGN 2012). Data regarding the medication profile will include any respiratory or allergy drugs added or removed, existing drug dosage changes, and drugs initiated for respiratory infections.

Patient inhaler technique will be evaluated by reviewing each video from the first 2 days of the intervention period and then a sample (at least one weekly) of the videos that have been uploaded to the repository application. The presence/absence of every required video will be documented.

Inhaler technique will be assessed and categorized on a three-point scale as effective, partially effective or poor (Patterson *et al.*, 2005). Inhaler technique evaluation will be based on criteria relevant to the child’s inhaler manufacturer administration directions, such as removal of cover, priming of device, vigorous shaking before each actuation, respiratory expiration before actuation, and holding of breath after inspiration. Effective technique will be recorded when the participant meets all relevant criteria. Partially effective technique will be recorded when errors in technique are observed but it is thought that the participant would receive some medication. Poor inhaler technique will be recorded when critical errors in technique occur such that it is considered unlikely that any medication would be inhaled (Patterson *et al.*, 2005; Melani *et al.*, 2011).

Adherence will be calculated based on total number of videos submitted during the 6 week intervention divided by the number of days during which DOT monitoring has occurred.

**Feedback questionnaires**

Parents/guardians will be asked to respond to feedback questionnaire to provide their views and experience about the use of the technology at the end of intervention (6 weeks) or in cases where a participant is unable to complete the study. Feedback questionnaires will be sent to each participant by the post and each participant will be contacted by telephone for a reminder if the questionnaire is not returned within a two week period. This feedback will be used to document participant’s experience or record any difficulties with using the mobile DOT approach in the study. Moreover, participants feedback will help us make improvements to the approach for the future. Two feedback questionnaires have been prepared by the research team: one for participants who completed the 6 week period of using mobile DOT (Appendix 20) and the second for participants who consented to take part but did not upload videos or who uploaded videos for a shorter period that the 6 weeks requested (Appendix 21).

**Data protection**

All participants’ data will be coded and entered into a database, using a unique study ID number, on the University’s firewalled server. The same approach of using study IDs will be applied to the video repository. Access to the custom designed, study specific repository, to be created in DropBox® will be password protected such that only researchers can access all files within the system, while study participants will only be able to access files which they have loaded themselves (again password protected). Hard copies of consent forms will be stored in a locked cabinet within the Clinical and Practice Research Unit in the School of Pharmacy, Queen’s University Belfast.

**Outcomes measure**

The primary outcome measures in this pilot study concern two areas. Firstly, the feasibility, practicality, and persistence of using a mobile DOT approach in the management of asthma in children and young people will be assessed by evaluation of ICS inhaler technique and monitoring inhaler use adherence. Secondly, asthma control associated with the mobile DOT approach will be assessed with clinical (including number of patients who progress to controlled category) and laboratory disease assessment, participant-reported medication adherence, and quality of life measurements (PAQOLQ/PACQOLQ and ACT/C-ACT).

Secondary outcome measures for the study include any impact of mobile DOT on participants’ inhaler technique and adherence. These two aspects of asthma management will be assessed by comparisons between intervention groups and within groups during, and across, phases of the study. Participant and parent/guardian factors that influence the impact of the DOT method on inhaler use and adherence also will be determined.

**Data analysis**

Statistical analyses will be conducted by the researcher after entering all data into SPSS (IBM SPSSv.23, USA). All nominal data collected during the study will be analysed using descriptive approaches. Group differences (I-med and D-med groups) will be studied using the independent sample Student t-test. If the data are not normally distributed non-parametric statistics will be utilised (Mann-Whitney U test for independent group differences). A paired Student t-test will be used to detect any differences associated with the study periods within each group

Adherence to ICS use in participants will be determined based on the patients’ general practitioner records of medicine prescribed and the patients’ records of medicine dispensed within the 6 week period, monitoring of video submissions, as well as the total scores achieved from the MARS questionnaires.

Categorical variables associated with non-adherence will be analysed using Chi-squared analysis. If the expected frequency falls below 5, the Fisher’s exact test will be employed.

Factors that affect the adherence to ICS inhaler use will be determined using patient demographic and clinical data together with responses obtained through administration of BMQ, CES-D, MARS, PACQOLQ/PAQOLOQ and ACT/C-ACT questionnaires to the participants and participants’ parents/guardians as appropriate. Multiple regression analysis will be performed to evaluate the relative contribution of different predictors to non-adherence.

**Dissemination of findings**

On completion of the study, the research team will prepare a full report on the findings covering all major aims and objectives of the study. Outcomes of the research will be submitted for publication in the pharmaceutical and medical literature. Results of the study will be presented at conferences about asthma care and/or children’s health care.

A short summary of the study findings in lay language will be provided to parents/guardians and/or participants at the conclusion of the project. The study will also form the basis of a PhD thesis.

**References**

AL-Jahdali H, Ahmed A, AL-Harbi A, *et al.*(2013) Improper inhaler technique is associated with poor asthma control and frequent emergency department visits. Allergy, Asthma & Clinical Immunology, 9(1):8.

Anagnostoua K., Harrison B., Ilesc R., Nasse S.(2012) Risk factors for childhood asthma deaths from the UK Eastern Region Confidential Enquiry 2001-2006. Primary Care Respiratory Journal, 21(1): 71-77.

Armstrong ML, Duncan CL, Stokes JO, Pereira D. (2014) Association of caregiver health beliefs and parenting stress with medication adherence in pre-schoolers with asthma. Journal of Asthma 51(4): 366-372. doi:10.3109/02770903.2013.876431

Bender B, Wamboldt FS, O'Connor SL, *et al.*  (2000) Measurement of children's asthma medication adherence by self report, mother report, canister weight, and Doser CT. Annals of Allergy, Asthma, & Immunology, 85(5):416-421

Bisgaard H. (1997) Delivery of inhaled medication to children. Journal of Asthma,34(6):443-467.

Brackis-Cott E, Mellins CA, Abrams E, Reval T, Dolezal C. (2003) Pediatric HIV medication adherence: The views of medical providers from two primary care programs. Journalof Pediatric Health Care 17:252-260.

British Thoracic Society and Scottish Intercollegiate Guidelines Network (SIGN). (2012) British guideline on the management of asthma. A national clinical guideline. (SIGN publication no. 101).<https://www.brit-thoracic.org.uk/guidelines-and-quality-standards/asthma-guideline/> Accessed 21 July, 2014.

BryantL., Bang C., Chew C., Baik SH., Wiseman D.(2013) Adequacy of inhaler technique used by people with asthma or chronic obstructive pulmonary disease, J PRIM Health care,5(3):191–198.

Cohen J, MannDM, Wisnivesky JP, *et al.*(2009). Assessing the validity of self-reported medication adherence among inner-city asthmatic adults: the Medication Adherence Reporting Scale for asthma. Annals of Allergy, Asthma & Immunology 103(4):325-331.

Creary SE., Gladwin MT., Byrne M., Hildesheim M., Krishnamurti L. (2014) A pilot study of electronic directly observed therapy to improve hydroxyurea adherence in pediatric patients with sickle-cell disease. Pediatric Blood &Cancer, 61(6):1068-1073.

DeMaio J, Schwartz L, Cooley P,Tice A. (2001) The application of telemedicine technology to a directly observed therapy program for tuberculosis: A pilot project. Clinical Infectious Disease 33, (12):2082-2084.

Dosumu EA. Compliance in pulmonary tuberculosis patients using directly observed treatment short course.African Journal of Medicine and Medical Sciences,30(1-2):111-114.

Drotar D, Bonner MS., (2009) Influences on adherence to paediatric asthma treatment: a review of correlates and predictors. Journal of Development & Behavioral Pediatrics 30(6): 574–582

Favorov M, Belilovsky E, Aitmagambetova I, Ismailov S, White ME, Chorba T. (2010) Tuberculosis deaths averted by the implementation of the DOTS strategy in Kazakhstan. The International Journal of Tuberculosis and Lung Disease, 14(12):1582-1588

Fish L, Lung CL. (2001) Adherence to asthma therapy. Annals of Allergy, Asthma and Immunology, 68(6): 24-30.

Global initiative for asthma (GINA 2012).[Pocket Guide for Asthma Management and Prevention in Children and adult older than 5 Years](http://www.ginasthma.org/documents/3) . Available at [http://www.ginasthma.org/. Access](http://www.ginasthma.org/.%20%20Access) Date (20/01/2014).

Gore SM. (1981) Assessing clinical trials- restricted randomisation. British Medical Journal (Clinical research ed.), 282(6282):2114-7.

Gupta R, Sheikh A, Strachan DP, Anderson HR. (2004) Burden of allergic disease in the UK: secondary analyses of national databases. Clinical &Experimental Allergy, 34 (4): 520–526

Horne R, Weinman J, and Hankins M (1999) The beliefs about medicines questionnaire: The development and evaluation of a new method for assessing the cognitive representation of medication. Psychology and Health, 14(1):1-24.

Horne R, Weinman J. (2002) Self-regulation and self-management in asthma: exploring the role of illness perceptions and treatment beliefs in explaining non-adherence to preventer medication. Psychology and Health, 17(1):17-32.

Horne R, Hankins M. (2008)*The medication adherence report scale (MARS): a new measurement tool for elicting patients’ reports of non-adherence.* The School of Pharmacy, University of London, London, UK.

Hedlin G. (2014) Management of severe asthma in childhood – state of the art and novel perspectives. Pediatric Allergy and Immunology, 25(2):111-121.

Hedlin G, Konradsen J, Bush A. (2012) An update on paediatric asthma.EuropeanRespiratory Review,21(125):175-185.

International ERS/ATS guidelines ondefinition, evaluation and treatment of severe asthma (2014).EuropeanRespiratory Journal, 43:343–373

Juniper EF, Guyatt GH, Feeny DH, Ferrie PJ, Griffith LE, Townsend M. (1996) Measuring quality of life in children with asthma. Quality of Life Research,5(1):35-46.

Klot T, Brand PL, Bromhof-Roordink H, Duiverman E, Kaptein A. (2011) Parental illness perceptions and medication perception in childhood asthma,a focus group study. ActaPaediatrica, 100(2):248-252

Klok T, Kaptein AA, Duiverman EJ, Brand PL. (2014) It’s the adherence, stupid (that determines asthma control in preschool children)!.EuropeanRespiratory Journal, 43(3):783-791.

Liu AH, Zeiger R, Sorkness C, *et al.*(2007) Development and cross-sectional validation of the Childhood Asthma Control Test. Journal of Allergy and Clinical Immunology,119(4):817–825.

Maslan J, Mims JW. (2014) What is asthma? pathophysiology, demographics, and health care costs. Otolaryngologic Clinics of North America, 47(1):13–22.

Massingham K, Fox S, Smaldone A. (2014) Asthma therapy in pediatric patients: A systematic review of treatment with montelukast versus inhaled corticosteroids. Journal of Pediatric Health Care, 28(1):51–62.

Machira EPM,Obimbo EM, Wamalwa D, Gachare LN.(2011) Assessment of inhalation technique among asthmatic children and their carers at the Kenyatta National Hospital, Kenya. African Journal of Respiratory Medicine,7(1).

Melani AS, Bonavia M, Cilenti V, *et al.* (2011) Inhaler mishandling remains common in real life and is associated with reduced disease control. *Respiratory Medicine*, *105*(6):930-938.

Nathan RA, Sorkness CA, Kosinski M, et al. (2004) Development of the asthma control test: a survey for assessing asthma control. *Journal of Allergy and Clinical Immunology*, *113*(1): 59-65.

National Asthma Council Australia. How to use video (2014), Available at [www.nationalasthma.org.au/](http://www.nationalasthma.org.au/).

Nagakuma P, Thomas H. (2013) management of severe asthma in children. Paediatrics and Child Health, 23(7):291-295.

Osterberg L, Blaschke T. (2005) Adherence to Medication. New England Journal of Medicine, 353(5):487-497.

Otu A. (2013) Is the directly observed therapy short course (DOTS) an effective strategy for tuberculosis control in a developing country?. Asian Pacific Journal of TropicalDisease,3(3):227–231. 

Pasipanodya JG, Gumbo T. (2013) A meta-analysis of self-administered versus directly observed therapy effect on microbiologic failure, relapse, and acquired drug resistance in tuberculosis patients. Clinical Infectious Disease, cit167. Available at <http://cid.oxfordjournals.org/content/early/2013/03/05/cid.cit167.full.pdf+html>. Accessed 18-07-2014

Patterson EE, Brennan MP, Linskey KM, Webb DC, Shields MD, Patterson CC. (2005). A cluster randomised intervention trial of asthma clubs to improve quality of life in primary school children: the School Care and Asthma Management Project (SCAMP). Archives of Disease in Childhood, 90(8):786-791.

Price D, Bosnic-Anticevich S, Briggs A, *et al.* (2013) Inhaler competence in asthma: common errors, barriers to use and recommended solutions. Respiratory Medicine, 107(1):37-46.

Radloff LS. (1977) The CES-D scale. A self-report depression scale for research in the general population. Applied Psychological Measurement, 1(3):385-401.

Singh A, Wilkinson S, Braganza S. (2014). Smartphones and pediatric apps to mobilize the medical home. Journal of Pedriatrics05.037 (in press). Available at http://dx.doi: 10.1016/j.jpeds.2014.05.037. Accessed 21-07-2014

Strunk RC, Bacharier LB, Phillips BR, *et al.* (2008). Azithromycin or montelukast as inhaled corticosteroid–sparing agents in moderate-to-severe childhood asthma study. Jorunal of Allergy andClinical Immunology, *122*(6):1138-1144.

Sumino K, Cabana M.(2013) Medication adherence in asthma patient. Current Opinion in Pulmonary Medicine, 19(1):49-53.

Task force report ERA/ATA guideline on severe asthma, international ERS/ATS guidelines on definition, evaluation and treatment of severe asthma. (2014) European Respiratory Journal, 43(2):343–373.

The Palo Alto Medical Foundation, asthma handout quick references.( 2014), Accessed (28 /04/2014).Available at <http://www.pamf.org/asthma/education/handouts.html>.

Virchow JC, Crompton GK., Dal Negro R, *et al.*(2008)Importance of inhaler devices in the management of airway disease.RespiratoryMedicine, 102(1):10–19.

Vasbinder EC, Janssens HM, Maureen PMH, Mölken RV, Dijk LV, Winter BCM et al.(2013) e-Monitoring of Asthma Therapy to Improve Compliance in children using a real-time medication monitoring system (RTMM): the e-MATIC study protocol, BMC MedicalInformaticsandDecisionMaking,13(1):383-10.

Available<http://www.biomedcentral.com/1472-6947/13/38>

Wade VA, Karnon J, Eliott JA, Hiller JE. (2012) Home videophones improve direct observation in tuberculosis treatment: A mixed methods evaluation. PLoS ONE 7(11): e50155. doi:10.1371/journal.pone.0050155

Walley JD, Khan MA, Newell JN, Khan MH. (2001) Effectiveness of the direct observation components of DOTS for tuberculosis: a randomized controlled trial in Pakistan. Lancet 357(9257): 664-669.

World Health Organization.Media centre, asthma, Available at <http://www.who.int/mediacentre>. (Access Date 20/05/2014)

World Health Organization. Treatment of tuberculosis guidelines, 4^th^ edition. Available at <http://www.who.int/tb/publications/2010/9789241547833/en/>.

(access date 18/07/2014)

**Young NL**, Foster AM, Parkin PC,et al. (2000) Assessing the efficacy of a school-based asthma education program for children: a pilot study. Canadian Journal of Public Health, 92(1):30–34.

**Appendices**

| Appendix 1 | Parent/guardian invitation letter |
| --- | --- |
| Appendix 2 | Parent/guardian information sheet |
| Appendix 3 | Information sheet for children (6-12years) |
| Appendix 4 | Information sheet for young people (13-16 years) |
| Appendix 5 | Parent guardian consent form |
| Appendix 6 | Children assent form |
| Appendix 7 | Data collection form |
| Appendix 8 | GP notification of study participation letter |
| Appendix 9 | GP request for prescribing records letter |
| Appendix 10 | Pharmacist request of dispensing record letter |
| Appendix 11 | MARS, Medication Adherence Report Scale (parent form) |
| Appendix 12 | MARS, Medication Adherence Report Scale (child form) |
| Appendix 13 | BMQ-specific, Beliefs about Medicine Questionnaire (parent form) |
| Appendix 14 | BMQ-specific, Beliefs about Medicine Questionnaire (child form) |
| Appendix 15 | Paediatric Asthma Caregiver Quality of Life Questionnaire(PACQOLQ) |
| Appendix 16 | Paediatric Asthma Quality of Life Questionnaire(PAQOLQ) |
| Appendix 17 | Asthma Control Test (ACT) |
| Appendix 18 | Childhood Asthma Control Test (C-ACT) |
| Appendix 19 | Centre of Epidemiologic Studies Depression Scale (CES-D) |
| Appendix 20 | Participant feedback and suggestions for improvement of the Mobile App to help with inhaler use in children |
| Appendix 21 | Feedback and suggestions for improvement of the Mobile App to help with inhaler use in children |
